# Functional analysis of macrophages in Behçet's disease

**DOI:** 10.1186/1546-0096-13-S1-O6

**Published:** 2015-09-28

**Authors:** H Nakano, Y Kirino, K Higashitani, M Takeno, A Ueda, Y Ishigatsubo

**Affiliations:** 1 Yokohama City University, Internal Medicine and Clinical Immunology, Yokohama, Japan; 2 Yokosuka City Hospital, Rheumatic Diseases Center, Yokosuka, Japan

## Introduction

Behçet's disease (BD) is an inflammatory disorder of unknown cause. The previous genome-wide association studies identified the associations between BD and several loci. Among them, *CCR1*, *MEFV*, and *IL10* encode genes highly expressed in macrophages, suggesting roles of macrophages in BD.

## Objectives

To evaluate functional differences of macrophages between BD and healthy controls (HC).

## Methods

We have differentiated peripheral monocytes into M1 or M2 macrophages under presence of either M-CSF or GM-CSF, cytokines involved in M2 or M1 macrophage polarizations, respectively. Real-time PCR, western blotting, ELISA, and flow cytometric analyses were performed to evaluate CD68, CD163, and heme oxygenase (HO)-1 expressions.

## Results

Expression of CD163, and numbers of M1 and M2 macrophages from BD are found to be similar compared with HC. HO-1 expression in sera and macrophages tend to be lower in BD.

## Conclusion

Lower HO-1 expression in BD suggests functional alteration of M2 macrophages in BD. Further experiments are required to elucidate mechanisms how M1 or M2 macrophages are involved in pathogenesis of BD.

